# Readiness for Telemedical Services in Patients With Cardiovascular Diseases: Cross-sectional Study

**DOI:** 10.2196/33769

**Published:** 2022-10-18

**Authors:** Barbara Kalańska-Łukasik, Aleksandra Gładyś, Tomasz Jadczyk, Monika Gruz-Kwapisz, Wojciech Wojakowski, Malgorzata Kowalska

**Affiliations:** 1 Department of Cardiology and Structural Heart Diseases Medical University of Silesia Katowice Poland; 2 Department of Epidemiology Faculty of Medical Sciences in Katowice Medical University of Silesia Katowice Poland; 3 International Clinical Research Center Interventional Cardiac Electrophysiology Group St Anne's University Hospital Brno Czech Republic

**Keywords:** telemedicine, readiness, patient-cardiologist contact, telehealth, acceptance

## Abstract

**Background:**

Telemedicine solutions, especially in the face of epidemiological emergencies such as the COVID-19 pandemic, played an important role in the remote communication between patients and medical providers. However, the implementation of modern technologies should rely on patients’ readiness toward new services to enable effective cooperation with the physician. Thus, successful application of patient-centric telehealth services requires an in-depth analysis of users’ expectations.

**Objective:**

This study aimed to evaluate factors determining readiness for using telehealth solutions among patients with cardiovascular diseases.

**Methods:**

We conducted a cross-sectional study based on an investigator-designed, validated questionnaire that included 19 items (demographics, health status, medical history, previous health care experience, expected telehealth functionalities, and preferred remote communication methods). Multivariate logistic regression was applied to assess the relationship between readiness and their determinants.

**Results:**

Of the 249 respondents, 83.9% (n=209) consented to the use of telemedicine to contact a cardiologist. The nonacceptance of using telemedicine was 2 times more frequent in rural dwellers (odds ratio [OR] 2.411, 95% CI 1.003-5.796) and patients without access to the internet (OR 2.432, 95% CI 1.022-5.786). In comparison to participants living in rural areas, city dwellers demonstrated a higher willingness to use telemedicine, including following solutions: issuing e-prescriptions (19/31, 61.3% vs 141/177, 79.7%; *P*=.02); alarming at the deterioration of health (18/31, 58.1% vs 135/177, 76.3%; *P*=.03); and arranging or canceling medical visits (16/31, 51.6% vs 126/176, 71.6%; *P*=.03). Contact by mobile phone was preferred by younger patients (OR 2.256, 95% CI 1.058-4.814), whereas older patients and individuals who had no previous difficulties in accessing physicians preferred landline phone communication.

**Conclusions:**

During a nonpandemic state, 83.9% of patients with cardiovascular diseases declared readiness to use telemedicine solutions.

## Introduction

Telemedicine, as an integral component of modern health care, has been used for nearly 30 years [1]. It offers a wide range of services based on remote communication between patients and clinicians for virtual care including education, monitoring, and therapeutic interventions [2,3]. Importantly, telehealth is recognized by professional medical societies such as the European Society of Cardiology and the American College of Cardiology [4], which emphasizes its role in everyday clinical practice. The emergence of new guidelines supports the implementation of virtual care for patients with cardiovascular diseases (CVD) [5], with additional focus on web-based medical education [6] and training for health care professionals (ie, undergraduate educational modulus in the field of telemedicine and telehealth licenses) [7,8]. The aforementioned examples illustrate the growing importance of telehealth, which has an impact on the health care ecosystem including patients, providers, and medical institutions.

Telemedicine covers a wide range of synchronous (live) and asynchronous (store and forward) services, including home telemonitoring, remote measurements of vital signs, medical consultations over a telephone (traditional landline and mobile phones), communication via email, and dedicated patient’s portals, as well as the implementation of voice technology and smart speakers [9,10]. However, telemedical care should be tailored to patients’ needs, reflecting the complex aspects of readiness and willingness to use medical technologies. Accordingly, user experience translates into the preferences of individuals whose health status is affected by remote care. This reasoning is why telemedicine should answer the needs of specific medical domains, in which patients with CVD constitute a heterogeneous group in terms of diseases as well as electronic literacy. Of note, there are smartphone-proficient older adults as well as patients with many comorbidities for whom being digitally connected might be challenging [11]. Furthermore, potential barriers associated with telehealth (geographic: limited internet access in rural areas; financial: expensive internet connection; and sociological: no face-to-face contact and a lack of equipment compatibility) may create real-world problems [12]. Accordingly, understanding patients’ capabilities and, consequently, readiness for the novel technology used in the telemedical practice is a crucial step toward the development of successful remote care programs. Notably, the COVID-19 pandemic created a kind of “telemedicine boom” that was caused by the forced need to implement temporary telemedicine solutions, which were used by almost 100% of US patients [13]. Therefore, to exclude bias and focus on long-term sustainable applications, it seems necessary to assess patients’ opinions toward virtual medical services during a nonpandemic state. In the aim to provide a basis for a successful patient-specific telehealth design, we evaluated factors affecting preferences and readiness for virtual care in patients with CVD.

## Methods

### Study Design

Between March 2019 and January 2020, 249 patients were enrolled in this epidemiological cross-sectional study, representing approximately 10% of the yearly hospitalized population at the Department of Cardiology and Structural Heart Diseases, Medical University of Silesia in Katowice, Poland. Respondents completed questionnaires while being assisted by a physician. The inclusion criterion was informed consent to participate in the study. Other factors, such as multiple morbidities, reasons for hospitalization, age, and gender, did not affect recruitment. The exclusion criteria were the lack of consent due to personal preferences or severe clinical condition. The patients were informed about the possibility to take part in the study on the first day of hospitalization.

### Instrumentation

To evaluate the willingness and preferences of patients with CVD toward telemedicine, a 19-item validated questionnaire was designed at the Department of Epidemiology, Medical University of Silesia in Katowice, Poland. A detailed description of the research tool was presented in a publication by Kowalska et al [14]. Briefly, the questionnaire included questions about sociodemographic data, medical history, and potential hindrances while contacting cardiologists remotely. The obtained results of the validation procedure confirmed the usefulness of the questionnaire as the key questions had high repeatability, ranging from 80% to 100% (Cohen κ statistics ranged from 0.419 to 0.920).

### Ethics Approval

Ethics approval for this study was received from the Bioethical Committee of the Medical University of Silesia in Katowice, Poland (KNW/0022/KB1/160/1617) on February 3, 2017.

### Statistical Analysis

Statistical analysis was performed using the Statistica software (version 13.0; Dell Software Inc). The missing values were removed from the final database. The qualitative variables were presented by frequency and percentage. Simple tests (chi-square or Fisher test) were used to assess the differences between independent groups of patients. In the interpretation of the results, *P* values <.05 were considered statistically significant. Finally, the relevant relationships between particular variables were verified in the multivariable analysis (logistic regression models with Hooke-Jeeves and quasi-Newton estimation). Only the statistically significant variables obtained in bivariate analyses were included in the models. The result section presents adequately the goodness of fit of the used model (confirmed by chi-square test and its *P* value).

## Results

The total studied group included 249 patients aged 65.3 (SD 13.8) years; more than half (n=158, 63.5%) were male, and the majority (n=211, 84.7%) were city dwellers. The vast majority (n=209, 83.9%) of patients reported readiness for telemedicine solutions, whereas 34 (13.6%) patients were opposed, and 6 (2.4%) did not respond to the question. Further multivariate analysis was carried out on a group of 202 (81.1%) patients for which a complete set of answers was obtained.

Men and people with previous difficulties in accessing medical doctors more frequently declared readiness for telemedicine solutions (*P*=.006 and *P<*.001, respectively). The other independent variables had no statistically significant impact on readiness for telemedicine (Table 1). The analysis deliberately omitted race and ethnicity because the study group was homogeneously of the White race.

In comparison to participants living in rural areas, city dwellers demonstrated a higher willingness to use telemedicine solutions in a particular form such as issuing e-prescriptions (19/31, 61.3% vs 141/177, 79.7%; *P*=.02); alarming at the deterioration of health (18/31, 58.1% vs 135/177, 76.3%; *P*=.03); and arranging or canceling medical visits (16/31, 51.6% vs 126/176, 71.6%; *P*=.03). Furthermore, a significant correlation was found between the level of education and willingness to use specific telehealth services; patients reporting a secondary level of education showed almost complete (58/64, 90.6%) compliance with solutions to control blood pressure, temperature, and bodyweight (*P*=.03), as well as e-prescriptions service (*P*=.01). In turn, respondents without internet access showed the lowest interest in arranging or canceling medical visits (39/67, 58.2% vs 102/138, 73.9%; *P*=.02) or issuing e-prescriptions (44/67, 65.7% vs 115/139, 82.7%; *P*=.006; Table 2).

**Table 1:** Frequency of positive patients’ declaration toward telemedicine according to particular determinants (*P* value in chi-square test).

Determinant			Positive patient declaration, n/N (%)		*P* value
**Sex**					.006
	Female	66/85 (77.6)			
	Male	142/157 (90.4)			
**Age**					.053
	Older (aged ≥68 years)	98/120 (81.7)			
	Younger (aged <68 years)	111/123 (90.2)			
**Place of residence**					.68
	City	177/205 (86.3)			
	Rural areas	31/37 (83.8)			
**Education**					.20
	Primary	88/108 (81.5)			
	Secondary	62/70 (88.6)			
	Higher	57/63 (90.5)			
**Internet access**					.16
	Yes	138/156 (88.5)			
	No	68/83 (81.9)			
**Previous difficulties in accessing medical doctors**					<.001
	Yes	123/131 (93.4)			
	No	85/109 (77.9)			

**Table 2:** Factors influencing the readiness for telemedicine services of patients with cardiovascular diseases (percentage of declaration and *P* value of chi-square test).

Independent variable		Telemedicine services accepted by patients											
		Remote contact with a cardiologist, n/N (%)	*P* value	Telemonitoring of vital signs (blood pressure, temperature, and bodyweight), n/N (%)	*P* value	Issuing e-prescriptions, n/N (%)	*P* value	Alarming health status deterioration, n/N (%)	*P* value	Scheduling and managing of medical visits, n/N (%)	*P* value	Medication reminder, n/N (%)	*P* value
**Sex**			.17		.30		.87		.08		.29		.18
	Female (n=90)	53/67 (79.1)		51/67 (76.1)		52/67 (77.6)		44/67 (65.7)		42/66 (63.6)		29/66 (43.9)	
	Male (n=158)	122/141 (86.5)		116/141 (82.3)		108/141 (76.6)		109/141 (77.3)		100/141 (70.9)		76/141 (53.9)	
**Previous difficulties accessing cardiologists**			.42		<.001		.22		.12		.99		.39
	Yes (n=133)	107/124 (86.3)		109/124 (87.9)		99/124		96/124 (77.4)		85/123 (69.1)		66/123 (53.7)	
	No (n=112)	69/84 (82.1)		58/84 (69)		61/84 (72.6)		57/84 (67.9)		58/84 (69)		40/84 (47.6)	
**Living with family**			.61		.81		.10		.38		.67		.30
	Yes (n=193)	141/168 (83.9)		135/168 (80.4)		133/168 (79.2)		122/168 (72.6)		114/167 (68.3)		83/167 (49.7)	
	No (n=53)	34/39 (87.2)		32/39 (82.0)		26/39 (66.7)		31/39 (79.5)		28/39 (71.8)		23/39 (58.9)	
**Age**			.64		.89		.18		.40		.22		.21
	Younger (aged <68 years; n=126)	93/109 (85.3)		88/109 (80.7)		88/109 (80.7)		83/109 (76.1)		79/109 (72.5)		51/109 (46.8)	
	Older (aged ≥68 years; n=123)	83/100 (83)		80/100 (80)		73/100 (73)		71/100 (71)		64/99 (64.6)		55/99 (55.6)	
**Place of residence**			.10		.16		.02		.03		.03		.06
	City (n=211)	155/177 (85.9)		145/177 (81.9)		141/177 (79.7)		135/177 (76.3)		126/176 (71.6)		95/176 (53.9)	
	Rural areas (n=37)	23/31 (74.2)		22/31 (70.9)		19/31 (61.3)		18/31 (58.1)		16/31 (51.6)		11/31 (35.5)	
**Educational level**			.79		.03		.01		.16		.26		.08
	Primary (n=111)	71/86 (82.6)		63/86 (73.3)		59/86 (68.6)		60/86 (69.8)		58/86 (67.4)		48/86 (55.8)	
	Secondary (n=73)	55/64 (90.6)		58/64 (90.6)		57/64 (89.1)		53/64 (82.8)		49/64 (76.6)		36/64 (43.7)	
	Higher (n=63)	50/58 (81)		47/58 (81)		44/58 (75.9)		41/58 (70.7)		36/57 (63.2)		35/57 (38.6)	
**Internet access**			.61		.71		.006		.04		.02		.62
	Yes (n=158)	118/139 (84.9)		113/139 (81.3)		115/139 (82.7)		108/139 (77.7)		102/138 (73.9)		69/138 (50)	
	No (n=87)	55/67 (82.1)		53/67 (79.1)		44/67 (65.7)		43/67 (64.2)		39/67 (58.2)		36/67 (53.7)	

Table S1 in Multimedia Appendix 1 presents the results of the multivariate analysis of the relationship between readiness for virtual care applications and demographic or socioeconomic determinants. Patients who had no previous difficulties in contact with a cardiologist were more than 3 times less likely to use telemedicine for vital signs measurement (odds ratio [OR] 3.596, 95% CI 1.681-7.690). The lack of acceptance for issuing e-prescriptions was 2 times more frequent in rural dwellers (OR 2.411, 95% CI 1.003-5.796) and in patients with no access to the internet (OR 2.432, 95% CI 1.022-5.786). Similarly, participants living in rural areas and individuals without internet connection were 2 times less likely to implement telemedicine for alarming the deterioration of health and for managing medical visits. On the contrary, city dwellers and patients with a lower level of education reported willingness to use telemedicine for medication reminder.

Table 3 presents patients’ declarations of readiness for communication modalities in particular groups of subjects defined by sociodemographic determinants. Patients’ preferences for face-to-face contact with physicians significantly differed based on the age of respondents; older individuals (aged ≥68 years) accepted this solution more frequently than younger participants (aged <68 years)—34.3% (34/99) versus 12.1% (13/107), respectively. Similarly, older people and patients with a lower level of education preferred contact by landline phone. Mobile phone contact was preferred by younger patients and individuals with access to the internet. Younger patients and individuals living in the city were more likely to use email and web services to contact cardiologists.

The results of the multivariate analysis revealed that particular opinions about telehealth communication solutions varied on different determinants (Table S2 in Multimedia Appendix 1).

**Table 3:** Factors influencing the readiness for telemedicine communication modalities of patients with cardiovascular diseases (percentage of declaration and *P* value of chi-square or Fisher test).

Independent variable		Preferred type of contact with physician									
		Face-to-face, n/N (%)	*P* value	Landline phone, n/N (%)	*P* value	Mobile phone, n/N (%)	*P* value	Email contact, n/N (%)	*P* value	Web page, n/N (%)	*P* value
**Sex**			.77		.98		.29		.01		.08
	Male (n=90)	14/66 (21.2)		35/66 (53)		51/66 (77.3)		7/66 (10.6)		4/66 (6.1)	
	Female (n=158)	32/139 (23)		74/139 (53.2)		116/139 (83.4)		36/139 (25.9)		19/139 (13.7)	
**Previous difficulties accessing cardiologists**			.20		.06		.049		.44		.42
	Yes (n=133)	32/123 (26)		72/123 (58.5)		105/123 (85.4)		28/123 (22.8)		12/123 (9.8)	
	No (n=112)	15/82 (18.3)		37/82 (45.1)		61/82 (74.4)		15/82 (18.3)		11/82 (13.4)	
**Living with family**			.62		.28		.78		.34		.30
	Yes (n=193)	39/165 (23.6)		90/165 (54.5)		133/165 (80.6)		36/165 (21.8)		20/165 (12.1)	
	No (n= 53)	8/40 (20)		18/40 (45)		33/40 (82.5)		6/40 (15)		3/40 (7.5)	
**Age**			<.001		.03		.003		<.001		.001^a^
	Younger (aged <68 years; n=126)	13/107 (12.1)		49/107 (45.8)		95/107 (88.8)		35/107 (32.7)		19/107 (17.8)	
	Older (aged ≥68 years; n=123)	34/99 (34.3)		60/99 (60.6)		72/99 (72.7)		8/99 (8.1)		4/99 (4)	
**Place of residence**			.68		.94		.52		.03^a^		.02^a^
	City (n=211)	41/175 (23.4)		92/175 (52.6)		143/175 (81.7)		40/175 (22.9)		23/175 (13.1)	
	Rural areas (n=37)	6/30 (20)		16/30 (53.3)		23/30 (76.7)		2/30 (6.7)		0/30 (0)	
**Educational level**			.39		.01		.29		<.001		<.001
	Primary (n=111)	23/88 (26.1)		49/88 (55.7)		68/88 (77.3)		7/88 (7.9)		1/88 (1.1)	
	Secondary (n=73)	15/63 (23.8)		40/63 (87.3)		55/63 (87.3)		18/63 (28.6)		10/63 (15.9)	
	Higher (n=63)	9/55 (16.4)		20/55 (36.4)		44/55 (80)		18/55 (32.7)		12/55 (21.8)	
**Internet access**			.047		.52		.005		<.001^a^		<.001^a^
	Yes (n=158)	25/135 (18.5)		69/135 (51.1)		117/135 (86.7)		41/135 (30.4)		23/135 (17)	
	No (n=87)	21/68 (30.9)		38/68 (55.9)		48/68 (70.6)		1/68 (1.5)		0/68 (0)	

^a^Result of Fisher test.

## Discussion

### Principal Findings

The main group of patients enrolled the study included individuals aged >65 years, and those participants largely accepted telemedicine solutions (83.9%). Importantly, our findings are in line with previous findings reported in the literature [15,16]. The observed upward trend in the acceptance and, consequently, the consent of older adults for the daily use of a wide range of telemedicine devices was strongly emphasized [12,16]. Importantly, factors such as technical adaptation (previous training and patients’ own experiences) is closely associated with the acceptance of telemedicine [17]. Therefore, gerontechnology (technology for aging populations) must be well designed and suited for health self-controlling [18], especially as improvement in health status is not always felt by patients using telemedicine services. As an example, patients with heart failure who received telehealth during the COVID-19 pandemic did not improve their health condition [19]. Thus, it is crucial to understand patients’ expectations and realistic opportunities to implement virtual care in clinical practice.

We report that patients who had no previous difficulties in accessing cardiologists were against using the home telemonitoring of vital signs (blood pressure and weight measurement). Interestingly, the results of studies conducted during the COVID-19 pandemic showed that remote monitoring is warranted, and the patient’s results at home (eg, a 6-minute walk test on a smart watch and the self-measurement of blood pressure) were comparable to those obtained during medical appointments [20,21]. Notably, the patients responded to our survey at a time when there was no epidemiological threat in Poland (before January 2020). Perhaps, the COVID-19 sanitary restrictions and severely limited access to health services leveraged patients’ support for telemedicine.

We observed that rural dwellers and patients without access to the internet were 2 times more frequently opposed to using telemedicine for issuing e-prescriptions. Given territorial sociodemographics, the juxtaposition of these 2 patient groups is not coincidental, especially in Poland where the division between rural and urban areas continues to matter (eg, limits concerning economy, education, and access to the internet) [22]. In Poland, only 50% of rural households have access to the internet, with an approximate rate of 70% in the European Union [23,24]. On the contrary, the study by Shemesh and Barnoy [20] conducted on Israel’s population proved that there are no significant sociodemographic differences in the use of mobile health apps. However, the authors strongly emphasized the specificity of this region (a technologically developed country with a high acceptance rate of innovation across the sociodemographic gradient) that could have impacted the results of the study [25].

In our study, there was a lack of acceptance for telemedicine solutions for the alarming the provider in the case of health status deterioration and in the case of arranging or canceling medical visits. The answers given in our survey, despite the effort put into explaining each option of telemedicine services (except self-testing), may be associated with a lack of understanding and awareness of the functions that a telemedicine device can perform. Moreover, the patients might have subconsciously chosen a lower number of possibilities in the survey, as they were scared of technological difficulties in handling a large number of applications.

We reported that medicine reminders are significantly more frequent in urban areas and in patients with lower levels of education. Many studies have proved that irregular adherence to drug use is an important problem, and the most common reason for failing to achieve a therapeutic effect is the drug’s spontaneous reduction, which occurs in about 30% to 35% of patients [26,27]. Nowadays, not only rural but also urban areas are significantly associated with a higher percentage of people with lower levels of education, which may constitute a higher need for self-control in their treatments [28]. As the previous study shows [7], a lower level of education does not lead to a lack of acceptance, but it is associated with even better satisfaction after the introduction of telemedicine services.

Patients’ preferences for direct contact with physicians are statistically significant and the most frequent in older people (aged ≥68 years). Patients who gained trust in medical personnel (physicians and nurses) were more willing to use telemedical devices as observed in the previous study [29]. Dario et al [18] reported that older patients tend to trust clinicians with whom they are already familiar. This finding is one of the reasons why during the COVID-19 pandemic, there was an urgent need to train doctors in the field of telemedicine, so that they would help their patients in servicing eHealth [30]. Trust in the doctor was crucial in the process of learning about new telecommunications.

Similarly, older people and patients with no previous difficulties accessing cardiologists prefer contact by landline phone. Traditional landlines, although currently not popular, are still used by older adults due to several advantages; for example, they are not sensitive to network coverage, are easy to use, and are cheaper in the case of international phone calls. Analogously, the previous studies show the importance of easy-to-use technology [16], whereas Scheibe et al [31] conclude that design features such as a simple intuitive menu, large icons, and high color contrast are especially important for older users. On the contrary, contact by mobile phone was preferred by younger patients and people who already have access to a cardiologist. Our findings are consistent with previous studies, in which young individuals were more eager to use telemedicine as they are more familiar with new technologies. Therefore, it could be concluded that experience in the use of mobile technologies, not age, is the main indicator of the willingness to use virtual care solutions [11].

### Limitations

The number of respondents participating in the study accounted for approximately 10% of the total number of patients hospitalized yearly at the Department of Cardiology, which is the main limitation of the presented results. However, the group of respondents is representatives of patients with CVD who statistically share common characteristics (age and gender). Furthermore, in the future, it is worth extending the survey to include the opinions of respondents who experienced telehealth during the COVID-19 pandemic and can verify which type of telemedicine solution is the most convenient.

### Conclusion

Patients with CVD are ready to accept remote solutions to contact with a cardiologist in clinical practice. They seem to be at least mostly aware of the needs and ready-made solutions that can make their everyday life easier. This finding confirms the fact that patients with CVD—mainly older adults—are often familiar with modern communication systems, which have become a natural component of daily life. Therefore, after identifying patients’ preferences associated with telehealth, the possibility of implementing user-friendly and well-designed telecommunication methods should be further explored.

## Appendix

Multimedia Appendix 1 Multivariate analyses.

## Abbreviations

CVD: cardiovascular diseases
OR: odds ratio
