# Supplementary material for: Readiness for Telemedical Services in Patients With Cardiovascular Diseases: Cross-sectional Study
Source: JMIR Form Res. 2022 Oct 18;6(10):e33769. doi: 10.2196/33769 (PMC11042508; doi:10.2196/33769)
Supplement: Multimedia Appendix 1 [file formative_v6i10e33769_app1.docx]

Table S1. Patient’s readiness for telemedicine solutions.

| **N=202** | **ANTICIPATED TELEMEDICINE SERVICES**  **Remote contact with a cardiologist (No agreement).Chi2( 7)=6.1283 p=0.5** | | | | | | | |
| --- | --- | --- | --- | --- | --- | --- | --- | --- |
|  | \| \| Intercept \| \| --- \| \| \| --- \| --- \| | \| Sex (Female =1) \| \| --- \| | \| Age \| \| --- \| | \| Place of residence (city=1) \| \| --- \| | \| Living with family (Yes=1) \| \| --- \| | \| Education (Primary = 1) \| \| --- \| | \| Internet access (Yes=1) \| \| --- \| | \| Previous difficulties in contact with physician (Yes=1) \| \| --- \| |
| \| Regression coefficient \| \| --- \| | -1.243 | -0.638 | 0.003 | 0.857 | -0.419 | -0.103 | -0.242 | 0.251 |
| \| Odds ratio \| \| --- \| | 0.288 | 0.528 | 1.003 | 2.356 | 0.658 | 0.902 | 0.785 | 1.285 |
| \| -95%CL \| \| --- \| | 0.010 | 0.231 | 0.971 | 0.887 | 0.203 | 0.537 | 0.282 | 0.580 |
| \| +95%CL \| \| --- \| | 8.055 | 1.208 | 1.036 | 6.256 | 2.129 | 1.516 | 2.184 | 2.847 |
| **Telemonitoring of vital signs (blood pressure, temperature, bodyweight)**  **(No agreement).Chi2( 7)=17.521 p=0.01** | | | | | | | | |
| Regression coefficient | -1.542 | -0.591 | -0.012 | 0.745 | -0.345 | -0.278 | -0.047 | 1.280 |
| \| Odds ratio \| \| --- \| | 0.214 | 0.554 | 0.988 | 2.107 | 0.708 | 0.757 | 0.954 | 3.596 |
| \| -95%CL \| \| --- \| | 0.010 | 0.254 | 0.959 | 0.807 | 0.250 | 0.462 | 0.370 | 1.681 |
| \| +95%CL \| \| --- \| | 4.751 | 1.211 | 1.019 | 5.502 | 2.001 | 1.241 | 2.459 | 7.690 |
| **Issuing e-prescriptions (No agreement). Chi2( 7)=14.679 p=0.04** | | | | | | | | |
| \| Regression  coefficient \| \| --- \| | -4.780 | 0.173 | -0.002 | 0.880 | 0.533 | -0.027 | 0.889 | 0.396 |
| \| Odds ratio \| \| --- \| | 0.008 | 1.189 | 0.998 | 2.411 | 1.703 | 0.974 | 2.432 | 1.486 |
| \| -95%CL \| \| --- \| | 0.000 | 0.558 | 0.969 | 1.003 | 0.727 | 0.624 | 1.022 | 0.745 |
| \| +95%CL \| \| --- \| | 0.180 | 2.535 | 1.028 | 5.796 | 3.990 | 1.519 | 5.786 | 2.964 |
| **Alarming health status deterioration (No agreement). Chi2( 7)=16.618 p=0.02** | | | | | | | | |
| \| Regression coefficient \| \| --- \| | -1.128 | -0.666 | -0.022 | 0.878 | -0.458 | 0.086 | 0.951 | 0.500 |
| \| Odds ratio \| \| --- \| | 0.324 | 0.514 | 0.978 | 2.405 | 0.632 | 1.090 | 2.588 | 1.649 |
| \| -95%CL \| \| --- \| | 0.022 | 0.256 | 0.952 | 1.018 | 0.249 | 0.710 | 1.101 | 0.848 |
| \| +95%CL \| \| --- \| | 4.871 | 1.029 | 1.004 | 5.682 | 1.607 | 1.674 | 6.080 | 3.210 |
| **Scheduling and managing of medical visits (No agreement). Chi2( 7)=13.305 p=0.06** | | | | | | | | |
| \| Regression coefficient \| \| --- \| | -1.908 | -0.349 | -0.009 | 0.895 | -0.482 | 0.302 | 0.909 | -0.018 |
| \| Odds ratio \| \| --- \| | 0.148 | 0.705 | 0.991 | 2.448 | 0.618 | 1.352 | 2.483 | 0.982 |
| \| -95%CL \| \| --- \| | 0.011 | 0.358 | 0.967 | 1.063 | 0.255 | 0.898 | 1.093 | 0.519 |
| \| +95%CL \| \| --- \| | 2.086 | 1.388 | 1.016 | 5.639 | 1.494 | 2.037 | 5.640 | 1.856 |
| **Medication reminder (Agreement). Chi2( 7)=18.131 p=0.01** | | | | | | | | |
| \| Regression coefficient \| \| --- \| | -0.370 | 0.559 | 0.023 | -0.983 | 0.484 | -0.384 | -0.224 | -0.305 |
| \| Odds ratio \| \| --- \| | 0.691 | 1.750 | 1.023 | 0.374 | 1.622 | 0.681 | 0.799 | 0.737 |
| \| -95%CL \| \| --- \| | 0.057 | 0.916 | 0.999 | 0.159 | 0.738 | 0.465 | 0.372 | 0.407 |
| \| +95%CL \| \| --- \| | 8.446 | 3.342 | 1.048 | 0.878 | 3.563 | 0.999 | 1.716 | 1.336 |

Table S2. Patient's preferred tools for communication with a medical doctor.

| **N=202** | **Face-to-face contact with medical doctor (Agreement). Chi2( 7)=19.155 p=0.007** | | | | | | | |
| --- | --- | --- | --- | --- | --- | --- | --- | --- |
|  | \| \| Intercept \| \| --- \| \| \| --- \| --- \| | \| Sex (Female =1) \| \| --- \| | \| Age \| \| --- \| | \| Place of residence (city=1) \| \| --- \| | \| Living with family (Yes=1) \| \| --- \| | \| Education (Primary = 1) \| \| --- \| | \| Internet access (Yes=1) \| \| --- \| | \| Previous difficulties in contact with physician (Yes=1) \| \| --- \| |
| Regression coefficient | -3.362 | 0.181 | 0.055 | -0.439 | -0.520 | -0.110 | 0.039 | -0.477 |
| \| Odds ratio \| \| --- \| | 0.035 | 1.199 | 1.057 | 0.644 | 0.595 | 0.896 | 1.039 | 0.621 |
| \| -95%CL \| \| --- \| | 0.001 | 0.551 | 1.021 | 0.226 | 0.224 | 0.563 | 0.430 | 0.298 |
| \| +95%CL \| \| --- \| | 1.060 | 2.609 | 1.094 | 1.839 | 1.581 | 1.427 | 2.511 | 1.295 |
|  | **Line phone (No agreement). Chi2( 7)=24.260 p=0.001** | | | | | | | |
| Regression coefficient | 0.453 | -0.054 | -0.049 | 0.221 | 0.386 | 0.226 | 0.463 | 0.646 |
| \| Odds ratio \| \| --- \| | 1.573 | 0.947 | 0.952 | 1.247 | 1.472 | 1.254 | 1.589 | 1.908 |
| \| -95%CL \| \| --- \| | 0.117 | 0.492 | 0.928 | 0.535 | 0.674 | 0.850 | 0.727 | 1.037 |
| \| +95%CL \| \| --- \| | 21.095 | 1.823 | 0.977 | 2.911 | 3.215 | 1.851 | 3.474 | 3.509 |
|  | **Mobile phone (No agreement). Chi2( 7)=15.037 p=0.03** | | | | | | | |
| Regression coefficient | -4.279 | -0.370 | 0.022 | 0.269 | -0.667 | 0.124 | 0.691 | 0.814 |
| \| Odds ratio \| \| --- \| | 0.014 | 0.691 | 1.023 | 1.308 | 0.513 | 1.133 | 1.996 | 2.256 |
| \| -95%CL \| \| --- \| | 0.000 | 0.312 | 0.988 | 0.470 | 0.180 | 0.690 | 0.779 | 1.058 |
| \| +95%CL \| \| --- \| | 0.433 | 1.531 | 1.058 | 3.638 | 1.467 | 1.858 | 5.114 | 4.814 |
|  | **E-mail contact (Yes, patient agree). Chi2( 7)=62.793 p<0.000001** | | | | | | | |
| Regression coefficient | 3.338 | 1.249 | -0.066 | -1.320 | 0.140 | 0.302 | -1.939 | 0.000 |
| \| Odds ratio \| \| --- \| | 28.149 | 3.487 | 0.936 | 0.267 | 1.151 | 1.352 | 0.144 | 1.000 |
|  | **Web page (Yes, patient agree). Chi2( 7)=43.927 p<0.000001** | | | | | | | |
| Regression coefficient | 43.883 | 0.587 | -0.044 | -22.726 | -0.090 | 0.778 | -23.688 | 0.650 |
| \| Odds ratio \| \| --- \| |  | 1.799 | 0.957 | 0.000 | 0.913 | 2.176 | 0.000 | 1.915 |
